# Characteristics and prognostic value of cardiac magnetic resonance strain analysis in patients with different phenotypes of heart failure

**DOI:** 10.3389/fcvm.2024.1366702

**Published:** 2024-05-17

**Authors:** Bianjie Zhao, Shiwen Zhang, Liang Chen, Kai Xu, Yinglong Hou, Shuguang Han

**Affiliations:** ^1^ Department of Nephrology, The Affiliated Xuzhou Municipal Hospital of Xuzhou Medical University, Xuzhou, Jiangsu, China; ^2^ Department of General Practice, The Affiliated Hospital of Xuzhou Medical University, Xuzhou, Jiangsu, China; ^3^ School of Medical Imaging, Xuzhou Medical University, Xuzhou, Jiangsu, China; ^4^ Department of Radiology, The Affiliated Hospital of Xuzhou Medical University, Xuzhou, Jiangsu, China; ^5^ Department of Cardiology, Shandong Provincial Qianfoshan Hospital, Shandong University, Jinan, China; ^6^ Department of Cardiology, The First Affiliated Hospital of Shandong First Medical University & Shandong Provincial Qianfoshan Hospital, Shandong Medicine and Health Key Laboratory of Cardiac Electrophysiology and Arrhythmia, Jinan, China

**Keywords:** heart failure, global longitudinal strain, global circumferential strain, global radial strain, prognosis

## Abstract

**Background:**

Strain analysis of cardiac magnetic resonance imaging (CMR) is important for the prognosis of heart failure (HF). Herein, we aimed to identify the characteristics and prognostic value of strain analysis revealed by CMR in different HF phenotypes.

**Methods:**

Participants with HF, including HF with reduced ejection fraction, HF with mildly reduced ejection fraction, and HF with preserved ejection fraction, and controls were enrolled. The baseline information and clinical parameters of participants were collected, and echocardiography and CMR examination were performed. Three-dimensional strain analysis was performed in the left ventricle, right ventricle, left atrium, and right atrium using CMR. A multifactor Cox risk proportional model was established to assess the influencing factors of cardiovascular adverse events in patients with HF.

**Results:**

During a median follow-up of 999 days (range: 616–1334), 20.6% of participants (73/354) experienced adverse events (HF readmission and/or cardiovascular death). Univariable Cox regression revealed that a 1% increase in left atrial global longitudinal strain (LAGLS) was associated with a hazard ratio (HR) of 1.21 [95% confidence interval (CI):1.15–1.28; *P* < 0.001]. Left ventricular global circumferential strain (LVGCS) (HR, 1.18; 95% CI: 1.12–1.24; *P* < 0.001), and left ventricular global longitudinal strain (LVGLS) (HR, 1.27; 95% CI: 1.20–1.36; *P* < 0.001) were also associated with HF hospitalizations and cardiovascular deaths. Among clinical variables, hypertension (HR, 2.11; 95% CI: 1.33–13.36; *P* = 0.002), cardiomyopathy (HR, 2.26; 95% CI: 1.42–3.60; *P* < 0.001) were associated with outcomes in univariable analysis. Multivariable analyses revealed that LAGLS (95% CI: 1.08–1.29; *P* < 0.001), LVGLS (95% CI:1.08–1.29; *P *< 0.001) and LVGCS (95% CI: 1.19–1.51; *P* < 0.001) were significantly associated with outcomes. Among clinical variables, hypertension (95% CI: 1.09–3.73; *P* < 0.025) remained a risk factor.

**Conclusion:**

CMR plays an obvious role in phenotyping HF. Strain analysis, particularly left atrial and left ventricular strain analysis (LAGLS, LVGLS, and LVGCS) has good value in predicting adverse outcome events.

## 1 Introduction

Heart failure (HF) is the end-stage stage of many cardiovascular diseases and has a high prevalence and mortality rate (1). HF with reduced ejection fraction (HFrEF) has been extensively studied with a compelling evidence base; however, there is a lack of similar data on HF with mildly reduced ejection fraction (HFpEF) and HF with midrange ejection fraction (HFmEF) (2). Most epidemiological and clinical trial data on HF are based on imaging with echocardiography; nonetheless, cardiac magnetic resonance imaging (CMR) is the gold standard for the majority of imaging parameters that comprise the latest guidance on HF and right ventricular assessment (3).

Myocardial strain is the deformation of the myocardium from the relaxed to the contracted state that can provide insight into the mechanics of the myocardium; it can be divided into three categories: global longitudinal strain (GLS), global circumferential strain (GCS), and global radial strain (GRS) (4). Myocardial strain analysis can reveal the deformation and velocity of the global and regional myocardium; it can be used as a developing indicator to assess the systolic and diastolic function. Compared with traditional parameters such as left ventricular ejection fraction (LVEF), myocardial strain analysis can analyze and assess the global and regional myocardial movement and function in detail at an early stage (5). CMR is a favorable tool for assessing the structure and function of the left ventricle (LV); it can serve as a gold standard for morphological structure and function assessment (6).

Left ventricular global longitudinal strain (LVGLS) is a broadly accepted strain index for evaluating the left ventricular global systolic function; it can predict hospitalization for HF and cardiovascular death (7, 8). GCS and GRS have not been well investigated in patients with HF. Although LV plays a significant role in controlling the contraction and relaxation of the heart, the importance of left atrial strain has gradually been paid attention to, and the research on it has also slowly increased (9). Furthermore, strain analysis of the right ventricle (RV) is increasingly being used in clinical practice. For example, compared with the ejection fraction of RV, strain analysis of RV can more sensitively identify early right ventricular dysfunction (10).

## 2 Materials and method

### 2.1 Study population

This retrospective, observational, cohort study was conducted at a single tertiary cardiac center (the Affiliated Hospital of Xuzhou Medical University, China) and was approved by the Institutional Review Board (Approval number: XYFY2021-KL116-01; May 25, 2021). The patients were recruited between July 2016 and September 2021, and they provided written informed consent during follow-up. The inclusion criteria for the HFpEF group and exclusion criteria for the study were consistent with those in our previous study (11). The inclusion criteria for the HFrEF and HFmEF group were as follows: (1) patients having typical HF signs and symptoms; (2) those with an N-terminal pro-brain natriuretic peptide level of >125 pg/ml; (3) those with LVEF between 41% and 49% were included in HFmEF group and those with LVEF of <40% were included in HFrEF group (12). Controls were recruited based on the following criteria: (1) be free of HF symptoms (2) LVEF ≥50% and be free of echocardiographic signs of severe diastolic dysfunction. Participants were excluded if they have: (1) Severe liver and kidney dysfunction (2). Severe heart valve disease (severe mitral stenosis, severe main stenosis) or known infiltrative or hypertrophic cardiomyopathy (3). Malignant tumors and severe hematological diseases (4). Severe infection (5). Autoimmune system diseases. All participants underwent a review of medical records and clinical data (general condition, medication, and blood sample analysis); transthoracic echocardiography and CMR were performed during the same visit.

### 2.2 Echocardiography acquisition and analysis

All participants underwent transthoracic echocardiography (iE33, Philips Healthcare, Best, The Netherlands), and cardiac systolic and diastolic function indices were obtained using a similar procedure used in our previous study (11).

### 2.3 CMR acquisition and analysis

A retrospectively gated cine-CMR method was utilized to capture cine images in cardiac short-axis, vertical long-axis, and horizontal long-axis orientations, while a steady-state free precession sequence was employed for volumetric analysis. The steady-state free precession images were utilized for cine imaging with specific parameters (repetition time msec/echo time msec, 3.2/1.2; flip angle, 64°; voxel size, 1.41.46 mm; matrix, 180 × 256 pixels).

CVI42 software (v5.13.5, Circle Vascular Imaging, Canada) was used by a single observer with over 3 years of experience to evaluate the CMR images, maintaining blindness to all data. Ventricular volumes, ejection fraction (EF), and left ventricular (LV) mass (excluding papillary muscles) were determined using balanced steady-state cine imaging and calculated from the short-axis cine stack. Endocardial borders were traced in end-systole and end-diastole, and volumes were averaged for calculation.

All volumetric and mass data were indexed to body surface area to derive ventricular volumetric index and mass index. The biplane approach was employed to calculate left atrial volumes, excluding the appendage and pulmonary veins, with maximum and minimum volumes determined. End-diastolic volume (EDV) and end-systolic volume (ESV) of the ventricular volumes were assessed from the volume-time curve for the maximal and minimal values to calculate EF.

Endocardial and epicardial borders of the LV, right ventricle (RV), and left atrium (LA) were delineated to enable semi-automatic tracking of the myocardium throughout the cardiac cycle. After automatic profile delineation, tracking performance was verified and manually adjusted if necessary to ensure accurate strain analysis. LA strain analysis utilized one two-chamber view and one four-chamber view. LV and RV strain were assessed using a stack of short-axis views and three long-axis views (two-chamber, three-chamber, and four-chamber views). GCS, GLS, and GRS values (GLS and GRS were obtained from LA strain analysis) were averaged from peak values of all 16 American Heart Association segments, excluding the most basal and apical sections. Tracking was repeated three times, and the average of these repetitions was used for further analysis, with repetitions conducted in separate sessions (13). The cardiologists who assessed outcomes of clinical outcomes and CMR parameters were all blinded to the group of patients.

Strain analysis was performed on LV, LA, and RV in the previous study, and in this study, we added the right atrium (RA). The delineation of endocardial and epicardial borders in RA was similar to that in LA; thus, only GLS and GRS were acquired from RA strain analysis.

### 2.4 Outcome data

All patients were followed up through telephone calls and interviewed by a single cardiologist in February 2022. The clinical endpoint was a composite of mortality or HF readmission (12). Assuming that recall may be inaccurate in some elderly patients, we conducted a secondary validation using hospital databases.

## 3 Statistical analysis

SPSS version 26.0 (IBM Corporation, Armonk, NY, USA) and GraphPad Prism Version 8.3.0 (GraphPad Software, San Diego, CA, USA) were used for statistical analyses. The mean + standard deviation (SD) or median (Q1, Q3) of continuous variables was calculated through a sample *t*-test in two groups. The categorical variables were expressed as numbers and percentages using the *χ*^2^ test. The Kaplan–Meier curves and univariable and multivariable Cox regression analyses were used to obtain the prognostic value of clinical and imaging risk factors.

## 4 Results

### 4.1 Baseline clinical characteristics

In total, 354 patients, including 143 with HFpEF, 43 with HFmEF, 82 with HFrEF, and 86 participants without HF, were enrolled. Table 1 shows the baseline characteristics of the study population, including demographics, clinical findings, functional status, medical history, medication history, and biochemistry results. Patients with HFpEF (58 ± 11 years) and HFmEF (56 ± 14 years) were older than the controls (44 ± 17 years). Patients with HFrEF had higher body mass index levels (26.5 ± 4.5 kg/m^2^) than the other patients. From the perspective of heart rate, it can be observed that as LVEF decreases, the heart rate of the subjects gradually increases. As LVEF decreases, blood pressure, especially SBP, tends to decrease. Compared to HFpEF subjects, HFmrEF and HFrEF subjects have a higher proportion of NYHA grade III or IV evaluations. Compared with the normal population, patients with HF have a higher proportion of previous coronary heart disease, myocardial infarction, hypertension, and cardiomyopathy. Moreover, they used more heart failure medication and anticoagulant drugs for maintaining or restoring cardiac function.

**Table 1:** Baseline clinical characteristics.

Characteristics	HFpEF *N *= 143	HFmEF *N *= 43	HFrEF *N *= 82	Control *N *= 86	*P* value
Demographics					
Age, years	58 ± 11^a,b^	56 ± 14^a,b^	47 ± 14	44 ± 17	<0.0001
Gender, *n* (%)					0.206
Male	95 (66.4)	22 (51.2)	57 (69.5)	54 (62.8)	
Female	48 (33.6)	21 (48.8)	25 (30.5)	32 (37.2)	
BMI, kg/m2	25.4 ± 3.9	25.8 ± 3.6	26.5 ± 4.5^a^	24.1 ± 3.2	0.001
Clinical findings					
HR, beats/min	78 ± 16^b,c^	85 ± 14^a^	85 ± 15^a^	74 ± 9	<0.0001
SBP, mmHg	128 ± 20	127 ± 25	129 ± 21	125 ± 14	0.469
DBP, mmHg	77 ± 12^b^	80 ± 18	85 ± 17^a^	77 ± 9	0.004
Smoking, *n* (%)	54 (37.8)^a,b^	23 (53.5)^a,b^	16 (19.5)^a^	3 (3.5)	<0.0001
Functional status					
NYHA, *n* (%)					
I/II	119 (83.2)^a,b^	36 (83.7)^a,b^	30 (36.6)^a^	NA	<0.0001
III/IV	15 (10.5)^a^	7 (16.3)^a^	46 (56.1)^a^	NA	<0.0001
Medical history					
CHD, *n* (%)	105 (73.4)^a^	33 (76.7)^a,b^	17 (20.7)	10 (11.6)	<0.0001
MI, *n* (%)	92 (64.3)^a^	29 (67.4)^a,b^	7 (8.5)	1 (1.2)	<0.0001
Hypertension, *n* (%)	71 (49.7)^a^	21 (48.8)^a^	30 (36.6)^a^	14 (16.3)	<0.0001
Cardiomyopathy, *n* (%)	17 (11.9)^b^	4 (9.3)^b^	50 (61.0)^a^	5 (5.8)	<0.0001
Hyperlipidemia, *n* (%)	15 (10.5)^b,c^	17 (39.5)^a^	8 (9.8)	5 (5.8)	<0.0001
T2DM, *n* (%)	28 (19.6)^a,b,c^	5 (11.6)^a^	9 (11.0)^a^	5 (5.8)	0.022
Medication					
Aspirin, *n* (%)	111 (76.6)^a,b^	30 (69.8)^a,b^	31 (37.8)	31 (36.0)	<0.0001
DAPT, *n* (%)	94 (65.7)^a,b^	27 (62.8)^a,b^	10 (12.2)	5 (5.8)	<0.0001
Statins, *n* (%)	116 (81.1)^a,b^	35 (81.4)^a,b^	30 (36.6)	36 (41.9)	<0.0001
β-blocker, *n* (%)	117 (81.8)^a^	35 (81.4)^a^	73 (89.0)^a^	30 (34.9)	<0.0001
ACEI/ARB, *n* (%)	95 (66.4)^a,b^	30 (69.8)^a,b^	77 (93.9)^a^	10 (11.6)	<0.0001
Diuretics, *n* (%)	47 (32.9)^a,b,c^	27 (62.8)^a,b^	76 (92.7)^a^	3 (3.5)	<0.0001
Blood biochemistry					
Hemoglobin, g/L	136 ± 18^b^	141 ± 18	147 ± 21	140 ± 14	0.001
Hematocrit, %	40.4 ± 6.3^b^	42.0 ± 5.5	4.38 ± 7.3	41.7 ± 3.8	0.007
eGFR, ml/min/1.73 m2	76.0 ± 51.6^a^	67.6 ± 18.9	80.1 ± 24.7^a^	60.7 ± 16.2	0.007
NT-proBNP, pg/ml Median (Q1, Q3)	1,579 (999, 2,564)^a,b,c^	3,093 (1,998, 5,449)^a^	241 (1,277, 3,849)^a^	5 (0, 51)	<0.0001
TG, mmol/L	1.5 ± 1.1^a^	1.5 ± 0.8	1.3 ± 0.6	1.1 ± 0.6	0.011
HDL, mmol/L	1.1 ± 0.3^a^	1.0 ± 0.2^a^	1.0 ± 0.2^a^	1.3 ± 0.4	<0.0001
Na+, mmol/L	140.5 ± 3.0^a^	139.3 ± 3.3^a,b^	140.9 ± 2.9	142.0 ± 2.3	<0.0001

Continuous variables were presented as mean ± SD or median (Q1, Q3). Category variables were presented as *n* (%). eGFR (estimated glomerular filtration rate) = 175 × creatinine^−1.234 ^× age^−0.179 ^× 0.79 (if female). *P* value < 0.05 was considered statistically significant. HFpEF, heart failure with preserved ejection fraction; HFmEF, heart failure with mid-range ejection fraction; HFrEF, heart failure with reduced ejection fraction; BMI, body mass index; HR, heart rate; SBP, systolic blood pressure; DBP, diastolic blood pressure; NYHA, New York Heart Association; CHD, coronary heart disease; MI, myocardial infarction; AF, atrial fibrillation; PAH, pulmonary arterial hypertension; T2DM, type 2 diabetes mellitus; DAPT, dual-anti platelet-therapy; ACEI, angiotensin converting enzyme inhibitors; ARB, angiotensin receptor blockers; CRP,C-reactive protein; NT-proBNP, N-terminal pro–B-type natriuretic peptide; HbA1c, glycated hemoglobin; TC, total cholesterol; TG, total triglyceride; HDL, high-density lipoprotein; LDL, low-density lipoprotein.

^a^ Significant difference compared to control.

^b^ Significant difference compared to HFrEF.

^c^ Significant difference compared to HFmEF.

### 4.2 Baseline imaging characteristics

The baseline imaging characteristics, including echocardiography and CMR measurements, are shown in Table 2. The ejection fraction of patients with different phenotypes of HF decreased to varying degrees (55.3 ± 5.6 for HFpEF, 46.5 ± 2.6 for HFmEF, 31.1 ± 5.3 for HFrEF, 62.5 ± 4.4 for controls), met the criteria. The CMR analysis of LV, RV, LA, and RA revealed the impairment of chambers in chamber volume and mass. Patients with HF had enlarged chamber volumes and larger chamber weights. These results remained unchanged after controlling for body mass index each strain was altered in the three groups compared with the control group. The mean values for left atrial GRS, left ventricular GRS, and left ventricular global circumferential strain (LVGCS) in the HFpEF group were different from that in the HFmEF group (*P* < 0.001). Moreover, all eight strains were altered in these groups compared with the HFrEF group (*P* < 0.001).

**Table 2:** Baseline imaging characteristics.

Characteristics	HFpEF *N *= 143	HFmEF *N *= 43	HFrEF *N *= 82	Control *N *= 86	*P* value
Echocardiography					
LVEF, %	55.3 ± 5.6^a,b,c^	46.5 ± 2.6^a,b^	31.1 ± 5.3^a^	62.5 ± 4.4	<0.0001
E/e’ ratio	15.0 ± 4.9	13.8 ± 7.6	16.0 ± 7.9	16.6 ± 7.1	0.538
CMR					
LV parameters					
LVEF, %	56.6 ± 10.5^a,b,c^	38.8 ± 17.2^a,b^	20.2 ± 11.4^a^	61.4 ± 7.1	<0.0001
LVEDVI, ml/m2	78.5 ± 26.07^b,c^	106.0 ± 38.8^b^	172.6 ± 247.7^a^	74.5 ± 16.9	<0.0001
LVESVI, ml/m2	35.5 ± 22.47^b,c^	68.1 ± 26.18^a,b^	115.4 ± 43.2^a^	28.2 ± 8.3	<0.0001
LVMI, g/m2	73.4 ± 25.5^a,b^	80.9 ± 36.9^a^	89.8 ± 24.8^a^	55.7 ± 12.7	<0.0001
RV parameters					
RVEF, %	47.5 ± 13.7^b,c^	40.2 ± 14.8^a,b^	23.6 ± 14.8^a^	49.0 ± 11.1	<0.0001
RVEDVI, ml/m2	72.7 ± 24.2	73.3 ± 18.2	78.7 ± 30.5	76.9 ± 20.5	0.375
RVESVI, ml/m2	39.8 ± 20.5^b^	45.6 ± 19.0^b^	78.7 ± 30.5^a^	38.4 ± 12.0	<0.0001
LA parameters					
LAVImin, ml/m2	21.2 ± 11.6^a^	22.5 ± 10.5^a^	32.3 ± 18.6^a^	13.0 ± 7.1	<0.0001
LAVImax, ml/m2	39.4 ± 18.4^a^	44.1 ± 14.4^a^	37.0 ± 24.1^a^	23.3 ± 17.6	<0.0001
LAGRS	22.07 ± 8.47^a,b,c^	17.95 ± 7.88^a,b^	13.22 ± 8.10*	26.42 ± 10.02	<0.0001
LAGLS	−12.80 ± 4.32^a,b^	−11.33 ± 4.75^a,b^	−7.55 ± 4.28^a^	−15.75 ± 4.23	<0.0001
LVGRS	17.96 ± 6.11^a,b,c^	11.17 ± 4.02^a^	9.85 ± 5.73^a^	20.86 ± 6.37	<0.0001
LVGLS	−11.39 ± 3.61^a,b^	−9.91 ± 3.53^a,b^	−6.51 ± 3.45^a^	−14.52 ± 3.42	<0.0001
LVGCS	−13.66 ± 3.28^a,b,c^	−9.80 ± 2.59^a,b^	−6.47 ± 4.06^a^	−15.90 ± 4.39	<0.0001
RAGRS	32.66 ± 17.60^a,b^	28.26 ± 13.82^a^	21.58 ± 12.66^a^	42.18 ± 19.88	<0.0001
RAGLS	−14.68 ± 6.09^a,b^	−13.60 ± 5.60^a^	−11.04 ± 5.08^a^	−17.16 ± 5.69	<0.0001
RVGRS	28.90 ± 13.41^b^	26.60 ± 9.95	20.42 ± 12.28^a^	30.72 ± 13.46	<0.0001
RVGLS	−14.86 ± 5.09^b^	−12.94 ± 5.37	−10.96 ± 4.97^a^	−15.27 ± 5.24	<0.0001
RVGCS	−9.33 ± 3.76^b^	−8.46 ± 4.28^b^	−6.12 ± 3.79^a^	−8.27 ± 3.96	<0.0001
Outcome data					
Follow-up(day)	846 ± 30^a,b^	793 ± 66^a,b^	1,109 ± 44	1,252 ± 43	<0.0001
Composite events	27	11	31	4	<0.0001

Data are presented in mean ± SD, *P* < 0.05 is considered statistically significant. LVEF, left ventricular ejection fraction; E/e’ ratio, mitral peak velocity of early filling (E) to early diastolic mitral annular velocity (e’); CMR, cardiovascular magnetic resonance; LVEDVI, left ventricular end- diastolic volume indexed to body surface area; LVESVI, left ventricular end-systolic volume indexed to body surface area; LVMI, left ventricular end-diastolic mass indexed to body surface area; RVEF, right ventricular ejection fraction; RVEDVI, right ventricular end-diastolic volume indexed to body surface area; RVESVI, right ventricular end-systolic volume indexed to body surface area; LAVImin, left atrium minimum volume indexed to body surface area; LAVImax, left atrium minimum volume indexed to body surface area; GCS, global circumferential strain; GLS, global longitudinal strain; GRS, global radial strain; LA, left atrium; LV, left ventricle; RA, right atrium, RV, right ventricle.

^a^ Significant difference compared to control.

^b^ Significant difference compared to HFrEF.

^c^ Significant difference compared to HFmEF.

### 4.3 Prognostic value of strain analysis in HF

**Figure 1:**
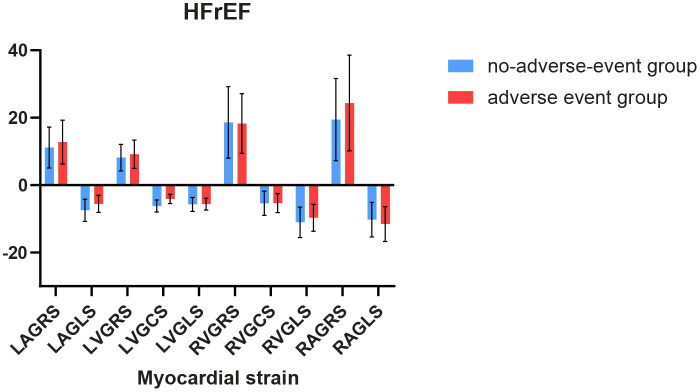
Comparison of myocardial strain between individuals with and without adverse events in HFmEF.

**Figure 2:**
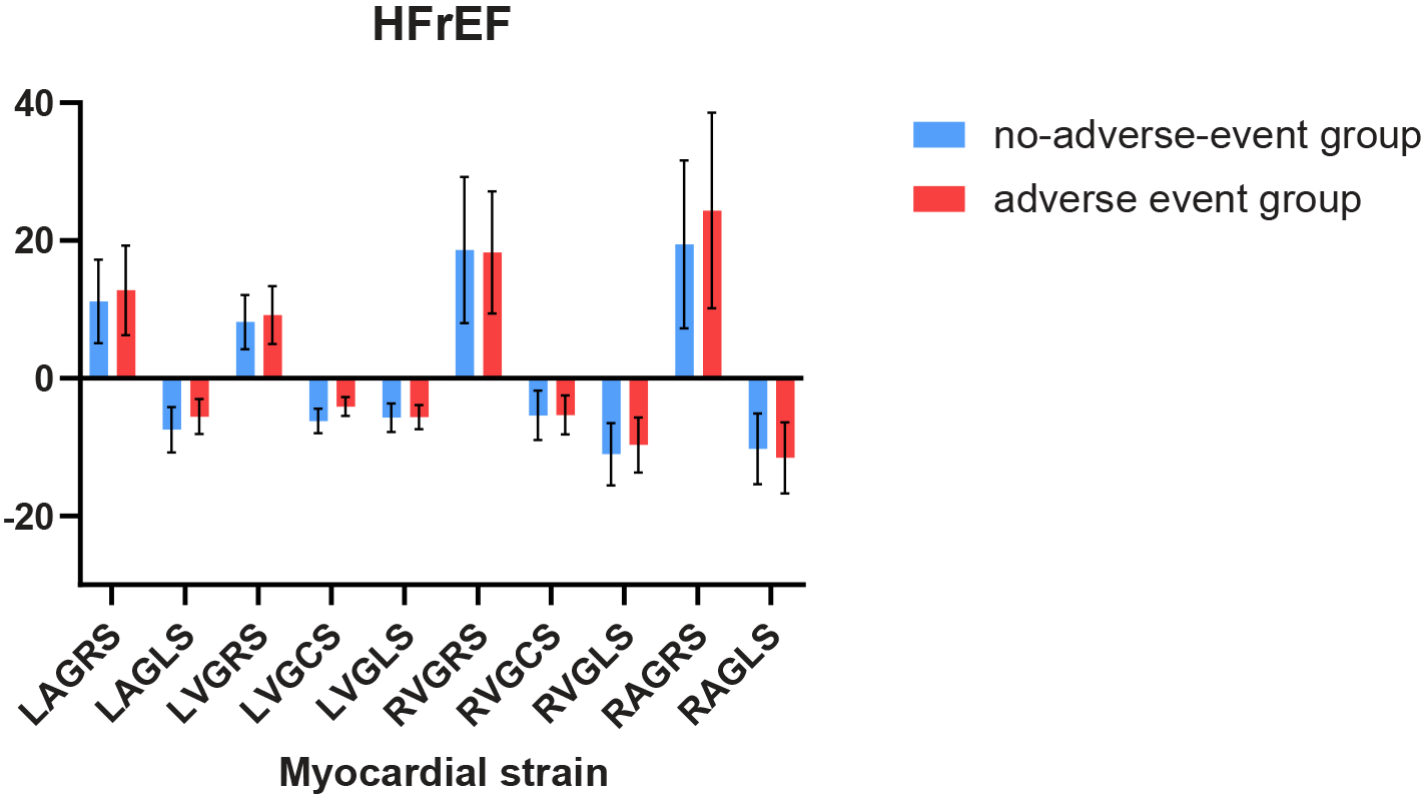
Comparison of myocardial strain between individuals with and without adverse events in HFrEF.

The follow-up was completed for all participants during a median follow-up of 999 days (616–1,334); 20.6% (73/354) of participants experienced adverse events, including HF readmission or cardiovascular death. Due to the serious confounding bias caused by the small sample size of HFmEF and HFrEF, regression analysis could not be implemented, but difference analysis was performed. The results are shown in Figures 1, 2. We performed COX regression analysis on HFpEF and obtained the results.

For 82 patients with HFrEF, LAGLS showed variation in any adverse event (*t* = 2.79, *P* = 0.007), similarly to LVGCS (*t* = 5.74, *P* < 0.001).Among the 43 patients with HFmEF in this study, we observed differences in LVGLS (*t* = 5.34, *P* < 0.001), LVGCS (*t* = 5.28, *P* < 0.001), and LAGRS (*t* = −4.41, *P* < 0.001) in relation to the endpoint events. The results are shown in Figures 1, 2.

In our study, the myocardial strain in the left ventricle and left atrium was associated with cardiovascular adverse events. After adjusting for other prognostic indicators, such as diabetes mellitus and smoking history, we found that the LAGLS (HR, 1.46; 95% CI: 1.07–1.46; *P* = 0.015), LVGRS (HR, 1.34; 95% CI: 1.07–1.68; *P* = 0.009), LVGCS (HR, 1.44; 95% CI: 1.06–1.94; *P* = 0.017), LVGLS (HR, 2.30; 95% CI: 1.513.49; *P* < 0.001) were all significantly associated with the occurrence of adverse cardiovascular events. The results are shown in Table 3.

**Table 3:** Univariate and multivariate Cox regression analysis of global longitudinal strain in HFpEF group.

HFpEF	Univariable analysis		Multivariable analysis	
Variable	Unadjusted hazard ratio	*P* value	Unadjusted hazard ratio	*P* value
Clinical variables				
Age, years	1.01 (0.98–1.05)	0.052		
NYHA	3.04 (0.98–9.40)	0.053		
Hypertension	0.96 (0.42–2.18)	0.930		
AF	0.60 (0.17–2.07)	0.421		
Cardiomyopathy	0.21 (0.07–0.21)	0.003		
CHD	2.89 (1.23–6.81)	0.015		
T2DM	1.14 (0.39–3.34)	0.801		
Smoking	3.61 (1.28–10.16)	0.015		
TC	0.98 (0.86–1.12)	0.862		
TG	0.88 (0.55–1.39)	0.594		
HDL	0.70 (0.16–3.04)	0.640		
LDL	1.08 (0.62–1.87)	0.778		
Cardiovascular MRI variables				
lagrs	0.93 (0.88–0.98)	0.004		
lagls	1.30 (1.15–1.46)	<0.001	1.46 (1.07–1.46)	0.015
lvgrs	1.94 (1.51–2.48)	<0.001	1.34 (1.07–1.68)	0.009
lvgcs	1.18 (1.12–1.24)	<0.001	1.44 (1.06–1.94)	0.017
lvgls	1.93 (1.51–2.48)	<0.001	2.30 (1.51–3.49)	<0.001
rvgrs	1.00 (0.97–1.03)	0.967		
rvgcs	1.02 (0.91–1.13)	0.757		
rvgls	1.06 (0.96–1.13)	0.285		
ragrs	0.99 (0.97–1.02)	0.882		
ragls	1.00 (0.93–1.07)	0.964		

Data in parentheses are 95% confidence intervals. *P* value < 0.05 was considered statistically significant. In this analysis, a predefined set of variables was entered into the multivariable model. The association was of strain at cardiovascular MRI and was adjusted for a predefined set of risk factors (age, clinical condition, global circumferential strain, global longitudinal strain, global radial strain) with the composite end point of heart failure hospitalization and cardiovascular death.

The univariate Cox regression analysis revealed that a 1% increase in left atrial global longitudinal strain (LAGLS) was associated with a hazard ratio (HR) of 1.21 [95% confidence interval (CI): 1.15–1.28; *P* < 0.001]. LVGCS (HR, 1.18; 95% CI: 1.12–1.24; *P* < 0.001) and LVGLS (HR, 1.27; 95% CI: 1.20–1.36; *P* < 0.001) were also associated with adverse events. Among clinical variables, hypertension (HR, 2.11; 95% CI: 1.33–13.36; *P* = 0.002) and cardiomyopathy (HR, 2.26; 95% CI: 1.42–3.60; *P* < 0.001) were associated with adverse outcomes.

In multivariable analyses, LAGLS (95% CI: 1.08–1.29; *P* < 0.001) and LVGCS (95% CI: 1.19–1.51; *P* < 0.001) were significantly associated with outcomes; the clinical variable hypertension (95% CI: 1.09–3.73; *P* < 0.025) remained a risk factor. Tables 3, 4 shows the results of univariate and multivariable Cox regression analyses. The results are shown in Table 4.

**Table 4:** Univariate and multivariate Cox regression analysis of global longitudinal strain for all populations.

Univariable analysis		Multivariable analysis		
Variable	Unadjusted hazard ratio	*P* value	Unadjusted hazard ratio	*P* value
Clinical variables				
Age, years	1.02 (1.00–1.04)	0.032		
NYHA	2.60 (1.63–4.16)	<0.001		
Hypertension	2.11 (1.33–3.36)	0.002	2.02 (1.09, 3.73)	0.025
AF	0.99 (0.47–2.07)	0.975		
Cardiomyopathy	2.26 (1.42–3.60)	<0.001		
CHD	1.21 (0.75–1.96)	0.440		
Smoking	1.41 (0.76–1.41)	0.263		
TC	0.99 (0.94–1.04)	0.765		
TG	1.12 (0.83–1.52)	0.434		
HDL	0.39 (0.14–1.03)	0.058		
LDL	1.04 (0.75–1.44)	0.773		
Cardiovascular MRI variables				
lagrs	0.94 (0.91–0.96)	<0.001		
lagls	1.21 (1.15–1.28)	<0.001	1.18 (1.08–1.29)	<0.0001
lvgrs	0.91 (0.88–0.94)	<0.001		
lvgcs	1.18 (1.12–1.24)	<0.001	1.34 (1.19–1.51)	<0.0001
lvgls	1.27 (1.20–1.36)	<0.001	1.18 (1.08–1.29)	<0.0001
rvgrs	0.98 (0.96–1.00)	0.061		
rvgcs	1.05 (0.99–1.12)	0.133		
rvgls	1.06 (1.01–1.11)	0.010		
ragrs	0.99 (0.97–1.00)	0.054		
ragls	1.03 (0.99–1.07)	0.146		

Data in parentheses are 95% confidence intervals. *P* value < 0.05 was considered statistically significant. In this analysis, a predefined set of variables was entered into the multivariable model. The association was of strain at cardiovascular MRI and was adjusted for a predefined set of risk factors (age, clinical condition, global circumferential strain, global longitudinal strain, global radial strain) with the composite end point of heart failure hospitalization and cardiovascular death.

Further receiver operating characteristic curve (ROC) analysis of LAGLS, LVGCS, and LVGLS showed that the critical value of LAGLS was −9.72 [area under the curve (AUC) 0.858, sensitivity 86.3%, specificity 78.6%], that of LVGCS was −12.43 (AUC 0.833, sensitivity 91.7%, specificity 61.2%), and that of LVGLS was −9.56 (AUC 0.866, sensitivity 93.1%, specificity 71.5%).

**Figure 3:**
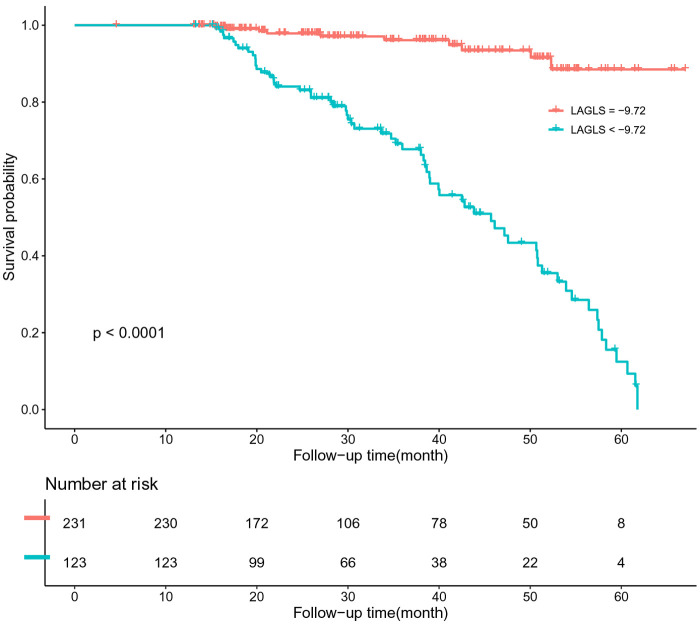
K-M survival curve based on ROC analysis of LAGLS critical values.

**Figure 4:**
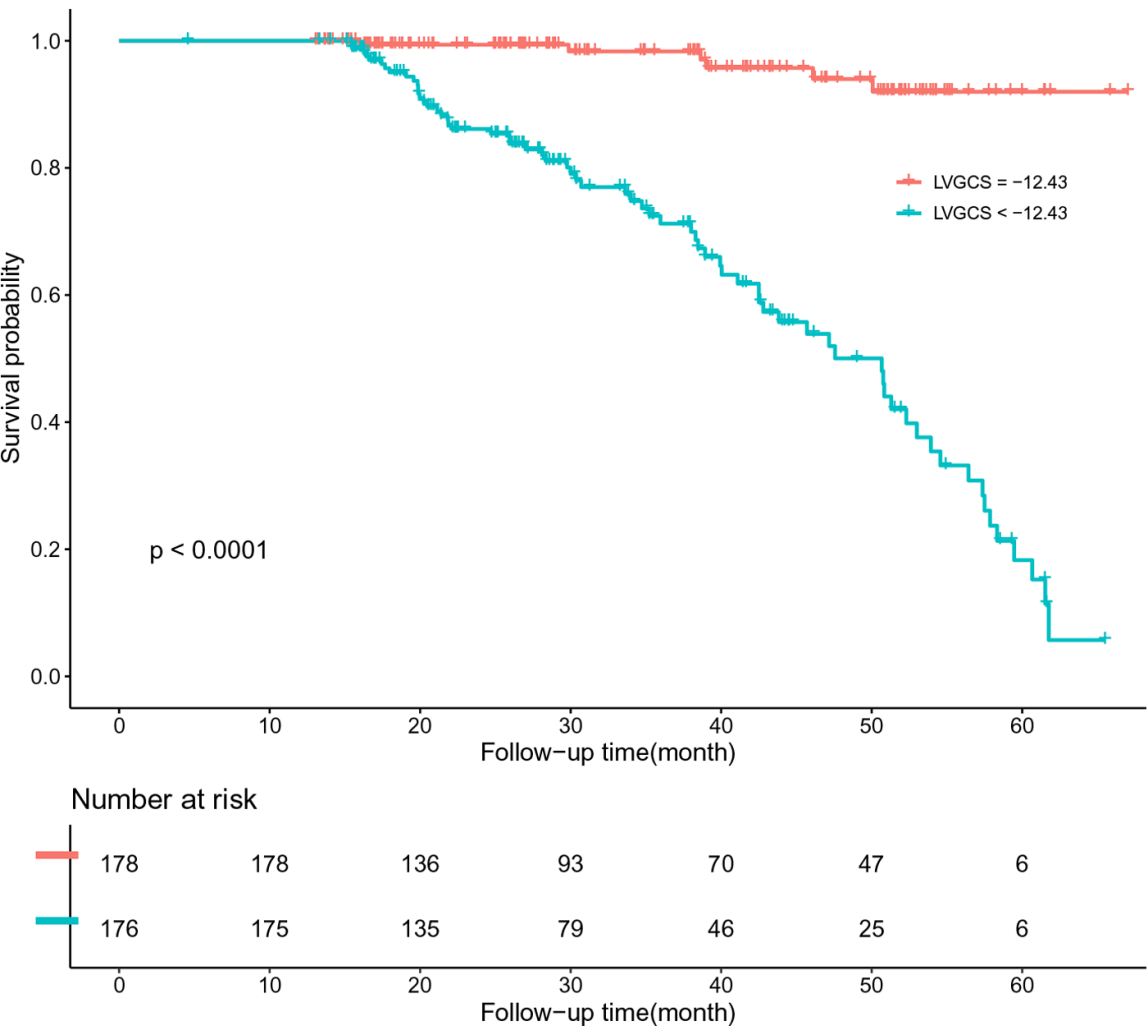
K-M survival curve based on ROC analysis of LVGCS critical values.

**Figure 5:**
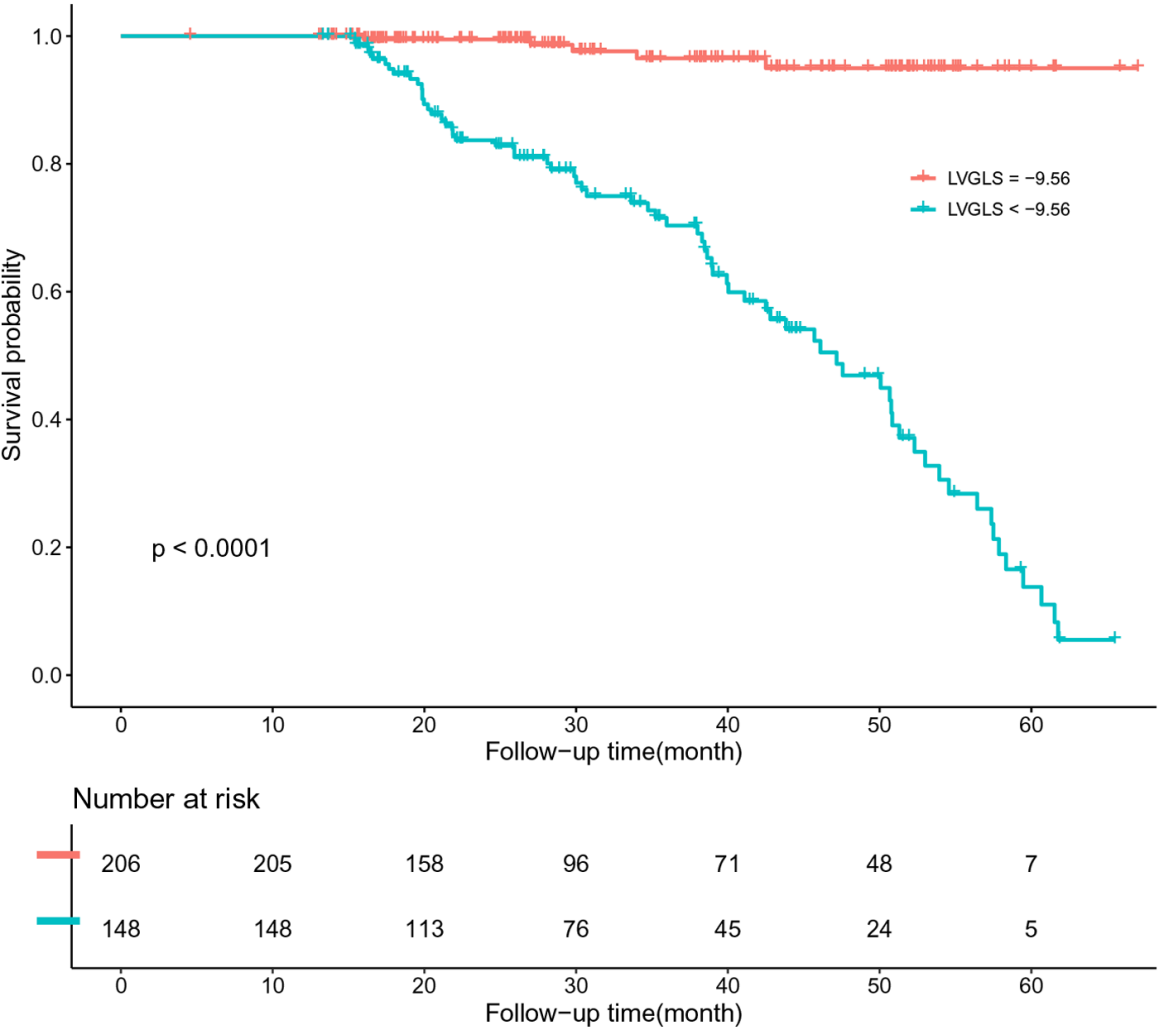
K-M survival curve based on ROC analysis of LVGLS critical values.

The patients were divided into groups according to the critical value. We drew the Kaplan–Meier survival curve of all patients in the group with a LAGLS of ≧9.72 and LAGLS of ≤9.72. The median survival time of patients with a LAGLS of ≧9.72 was 1,736 days (95% CI: 1,659–1,812) and that of patients with a LAGLS of ≤9.72 was 1,371 days (95% CI: 1,163–1,578). Significant differences were observed between the two groups (LogRank *P* < 0.001). Similarly, we drew a Kaplan–Meier survival curve with an LVGCS of 12.43 and an LVGLS of 9.56 as the critical value. The median survival time of the groups divided by the critical value was significantly different, and significant differences were observed between the groups (LogRank *P* < 0.001). The results are shown in Figures 3–5.

## 5 Discussion

CMR is the gold standard for cardiac evaluation, and its clinical application is also increasing recently. European guidelines classify HF based on left ventricular systolic function. However, the value of LVEF measured by echocardiography in evaluating the prognosis of HF patients is limited (14). Strain analysis can better identify different subtypes of heart failure and predict prognosis. In HFrEF, GLS, as a measure of left ventricular systolic function, is significantly associated with increased neurohormonal activation and early hemodynamic deterioration (15). In addition, some scholars have observed that RVGLS is an independent predictor of cardiac events in acute decompensated heart failure. Due to the small sample size in this study, we are currently unable to confirm that LAGLS and LVGCS can predict adverse events in HFrEF. However, the LAGLS and LVGCS values in the no-adverse-event group were significantly higher than those in the adverse-event group. Some scholars believe that patients with HFmEF are considered to have similar prognosis and clinical manifestations as patients diagnosed with HfpEF (16), but Strain analysis can better evaluate systolic function and predict prognosis. We found some indicators that may be related to prognosis in the HFmEF samples in this study, such as LVGLS, LVGCS, and LAGRS. Perhaps left atrial and left ventricular strain analysis has more advantages in predicting adverse events. Almost half of patients with signs and symptoms of heart failure have a normal ejection fraction on cardiac ultrasound, and this group of people needs more indicators to evaluate the prognosis. We found in HFpEF that LAGLS, LVGRS, LVGCS, and LVGLS are risk factors for adverse events in heart failure.

Although there have been many studies on CMR, most of them have been based on a single chamber, lacking visual presentation and comparison of each chamber (13, 17, 18). Therefore, we conducted this study to explore the association between each chamber and clinical practice.

By observing LA, we found that there were significant differences in the left atrial GRS among the four groups. Although LAGLS was not significantly different in the HFpEF and HFmEF groups, it was still different when compared with the control and HFrEF groups. Moreover, left atrial volume is closely associated with adverse events of heart disease. Benjamin H et al. found that strain analysis of LA provides significant value for the clinical diagnosis and prognosis of HFpEF (19).

We found similar results in LV, for example, LVGCS presented differently in four groups. Djawid Hashemi et al. found significant differences in LVGLS and LVGCS values by comparing HFrEF, HF with mildly reduced ejection fraction, and HFpEF, which is similar to our study results. This result may be related to the first injury of the left ventricular septum when HF occurs (20).

RV systolic dysfunction is a powerful predictor of mortality and HF-related hospitalization (21). By observing the myocardial strain force of RA and RV, we found that compared with the control group, the myocardial strain force of RA and RV had different degrees of change (including GRS, GLS, and GCS). However, compared with HFpEF and HFmEF, as well as HFmEF and HFrEF, we did not observe obvious strain change. This may be related to the fewer patients with HF caused by pulmonary hypertension in our study. J. L. Vos et al. confirmed that the myocardial strain of RV in HF caused by pulmonary hypertension was significantly damaged (22). Moreover, Louise A. E. Brown et al. observed 46 patients with HFmEF and 134 patients with HFpEF and found that patients with HFmEF and HFpEF had the most phenotypic characteristics, including the degree of microvascular injury (23).

To further study the relationship between myocardial strain and adverse events, we conducted a COX survival analysis. LAGLS, LVGCS, and LCGLS were significantly associated with adverse events in multivariate analysis. Further ROC analysis revealed that the critical values of LAGLS, LVGCS, and LVGLS were −9.72, −12.43, and −9.56, which will help doctors in clinical treatment and judgment of prognosis. It is noteworthy that there is a certain correlation between the left atrium and adverse cardiac events LAGLS has been proven to be a sensitive indicator for distinguishing hypertrophic cardiomyopathy from non-hypertrophic cardiomyopathy left ventricular hypertrophy (24). Some scholars found in a large cohort of patients that GCS has an increasing independent prognostic value besides the clinical variables LVEF and late gadolinium enhancement (25). Jian He et al. found that GRS and GLS are the indicators of early damage in HF caused by hypertension (26), which is similar to our study results. Therefore, they may play a certain role in the diagnosis of HF in the future.

Nevertheless, our study has several limitations. First, this was a retrospective study with a certain bias. Second, the follow-up time was less, which needs to be further extended; thus, the occurrence of end-point events was insufficient. Third, although strain analysis of RA was added, no valuable results were obtained. Finally, the clinical characteristics of patients with HF varied greatly, and the sample size of this study was small. In the future, multi-center and large-sample trials are warranted.

In conclusion, our study revealed the prognostic value of HF strain analysis and predicted the mean survival time according to the ROC curve. Strain analysis of LV and LA has a certain predictive value for positive events.

## Data Availability

The original contributions presented in the study are included in the article/Supplementary Material, further inquiries can be directed to the corresponding authors.
